# Dual targeting PET tracer [^68^Ga]Ga-PSFA-01 in patients with prostate cancers: A pilot exploratory study

**DOI:** 10.7150/thno.108676

**Published:** 2025-03-10

**Authors:** Yue Li, Lili Guan, Xiaoyang Zhang, Jia Li, Xinlin Wang, Wenbo Li, Lu Xu, Shuang Liu, Zhaobing Tang, Mengchao Cui, Hua Pang

**Affiliations:** 1 Department of Nuclear Medicine, The First Affiliated Hospital of Chongqing Medical University, No.1 Youyi Road, Yuzhong District, Chongqing, 400016, China.; 2 Key Laboratory of Radiopharmaceuticals, Ministry of Education, College of Chemistry, Beijing Normal University, Beijing, 100875, China.; 3 Department of Urology, The First Affiliated Hospital of Chongqing Medical University, No. 1 Youyi Road, Yuzhong District, Chongqing, 400016, China.

**Keywords:** [^68^Ga]Ga-PSFA-01, [^68^Ga]Ga-PSMA-11, [^68^Ga]Ga-FAPI-04, prostate cancer, PET/CT

## Abstract

**Purpose:** To assess the effectiveness of [^68^Ga]Ga-PSFA-01 PET/CT in detecting primary tumors and metastatic lesions in patients with prostate cancer (PCa), and to compare the results with those from [^68^Ga]Ga-PSMA-11 PET/CT and [^68^Ga]Ga-FAPI-04 scans.

**Methods:** Patients with histologically proven PCa were prospectively recruited and underwent [^68^Ga]Ga-PSFA-01 PET/CT, of which: 25 participants also underwent [^68^Ga]Ga-PSMA-11 PET/CT scan, 5 patients also underwent [^68^Ga]Ga-FAPI-04 PET/CT scan, 3 patients underwent three modalities imaging. To assess the expression of PSMA and FAP, we obtained a pathological tissue section from a patient and performed immunohistochemical staining analysis. SUV_max-PSFA_, SUV_max-PSMA_, SUV_max-FAPI_ and the number of detected lesions were compared by using the Wilcoxon signed-rank test, and the Mc-Nemar test was used to compare detectivity. Correlation between SUV_max-PSFA_ and prostate cancer related clinical indicators was demonstrated with Spearman's ratio. A visual assessment was made to compare the detectability of primary tumors and metastases in different regions.

**Results:** A total of 33 patients with a median age of 70 years (range: 52-89 years) were enrolled. Including 13 patients for initial staging and 20 for recurrence detection. [^68^Ga]Ga-PSFA-01 demonstrated superior performance in both patient-based and lesion-based analyses than [^68^Ga]Ga-PSMA-11 PET/CT. However, [^68^Ga]Ga-PSFA-01 depicted lower uptake in primary tumors (11.13 ± 7.04 vs. 15.44 ± 9.25, p = 0.009), bone metastases (8.50 ± 5.0 vs. 12.43 ± 9.55, p < 0.001) and metastases in other sites (6.05 ± 3.29 vs. 10.73 ± 8.74, p = 0.028) , lower tumor to background ratio (TBR) than [^68^Ga]Ga-PSMA-11 PET/CT (2.86 ± 1.50 vs. 9.50 ± 5.62, p < 0.001). [^68^Ga]Ga-PSFA-01 PET/CT showed more lesions (24 vs. 13, p = 0.18), higher uptake (primary tumors, 10.27 ± 2.42 vs. 7.32 ± 0.17, p = 0.109; bone metastases, 8.14 ± 5.98 vs.4.52 ± 1.22, p = 0.128; pelvic lymph nodes, 5.4 ± 2.83 vs.4.19 ± 1.39, p = 0.655) than [^68^Ga]Ga-FAPI-04 PET/CT. There was also a significantly positive correlation between SUV_max-PSFA_ of prostate lesions with the tPSA levels (r = 0.468, p = 0.016) and fPSA levels (r = 0.518, p = 0.04), a significantly negative correlation with the free-to-total prostate-specific antigen ratio (FPSAR) (r = -0.608, p = 0.012).

**Conclusion:** [^68^Ga]Ga-PSFA-01 PET/CT demonstrated higher detection rates and visual assessment efficacy compared to [^68^Ga]Ga-PSMA-11 PET/CT in PCa patients. While preliminary data suggest that [^68^Ga]Ga-PSFA-01 may also outperform [^68^Ga]Ga-FAPI-04 PET/CT, the sample size for [^68^Ga]Ga-FAPI-04 (n = 5) is limited, and further studies are needed to confirm these findings.

## Introduction

Prostate cancer (PCa) is the second most common cancer diagnosed in males and the fifth most prevalent cause of cancer-related deaths globally 1. Clinical manifestations of PCa are frequently absent at the time of initial diagnosis, which underscores the importance of proactive screening and early diagnosis 2. Consequently, achieving early and accurate initial staging, along with the implementation of personalized treatment strategies, remains a significant challenge in the management of PCa, which is crucial for improving prognosis. Prostate-specific antigen (PSA) has been a pivotal biomarker in the early detection of PCa, however, its diagnostic accuracy is limited, leading to a non-negligible rate of missed and incorrect diagnoses 3.

Prostate-specific membrane antigen (PSMA) is a type II transmembrane protein expressed in PCa cells. The use of radiopharmaceuticals targeting PSMA in positron emission tomography (PET) has become a preferred modality for assessing recurrence and has further applications in biopsy guidance, disease staging, and the evaluation of disease progression 4-8. However, the diagnostic accuracy of PSMA PET is challenged by evidence indicating that up to 15% of clinically significant PCa cases do not express this protein, resulting in false-negative outcomes 9-11. Therefore, the identification of alternative imaging biomarkers that can complement PSMA PET in these circumstances represents a significant unmet clinical need.

Over the past few years, the tumor microenvironment (TME) has gained significant attention due to its pivotal role in tumorigenesis, neo-angiogenesis, and the progression of cancer. The TME primarily consists of immune cells, extracellular matrix, vasculature, and cancer-associated fibroblasts (CAFs) 12. Fibroblast Activation Protein (FAP), a type II transmembrane serine protease, is almost nonexistent in benign tumors, healthy tissues, and normal fibroblasts (with the exception of chronic inflammatory situations), which are significantly overexpressed in CAFs 13. Overexpression of FAP elevates the risk of tumor invasion and lymph node metastasis in numerous solid cancers 14, including metastatic castration-resistant prostate cancer (mCRPC), particularly when the cancer exhibits neuroendocrine (NE) differentiation 15,16. In a study, the expression of FAP was compared with PSMA in 116 castration-resistant prostate cancer (CRPC) tumors. 11 of the 19 PSMA-negative tumors (58%) were found to be FAP-positive 17. In a treatment-naive PCa patient with low PSMA expression, [^18^F]-PSMA-1007 PET/CT showed low PSMA activity in the primary tumor and metastases, while [^18^F]-FAPI-04 PET/CT detected more positive lesions 18. These situations may include the inherent heterogeneity of PSMA expression in PCa, as well as the need to consider alternative imaging modalities when PSMA expression is low. FAP expression, which is dependent on tumor microenvironment pathways, might persist even when PSMA expression is reduced. Additionally, neuroendocrine differentiation in PCa can reduce PSMA expression, but may not affect FAP expression 19. Therefore, FAP could serve as a promising target for theranostic applications in PCa, especially with low or no PSMA expression 20.

As a clinically validated PSMA-targeted tracer, [^68^Ga]Ga-PSMA-11 has been widely used and has demonstrated high sensitivity and specificity in detecting PCa lesions. This tracer has been extensively studied and has shown significant impact on the management of PCa patients. It has favorable pharmacokinetics, with rapid uptake and clearance, which allows for high-quality imaging within a reasonable time frame 21. [^68^Ga]Ga-FAPI-04 has shown promising results in detecting metastatic lesions and providing complementary information to PSMA-targeted imaging 22. However, there are still several restrictions on the clinical application of single-target receptors. The expression levels of specific receptors may vary with the growth or differentiation of tumor cells. Given the heterogeneity of PCa and the need to improve tumor-targeting sensitivity in PET/CT imaging, Wang *et al.* synthesized a heterodimer with dual-targeting properties based on FAP and PSMA inhibitors, [^68^Ga]Ga-PSFA-01 23. In terms of chemical structure, [^68^Ga]Ga-PSFA-01 employed the targeting scaffold of [^68^Ga]Ga-PSMA-11 and [^68^Ga]Ga-FAPI-04, which makes it more appropriate to compare [^68^Ga]Ga-PSFA-01 with [^68^Ga]Ga-PSMA-11 and [^68^Ga]Ga-FAPI-04 in PET/CT imaging.

In this study, we aimed to further evaluate the clinical utility of [^68^Ga]Ga-PSFA-01 for depicting PCa and to compare the results with those from single-target tracers [^68^Ga]Ga-PSMA-11 and [^68^Ga]Ga-FAPI-04.

## Materials and Methods

### Patients

This prospective study was approved by the clinical research ethics committee of the First Affiliated Hospital of Chongqing Medical University (2024-048-01) and registered at ClinicalTrials.gov (NCT06387381), and all participants signed a written informed consent form. 33 patients with PCa were enrolled from March 2024 to October 2024 at the First Affiliated Hospital of Chongqing Medical University. The inclusion criteria were as follows: (a) having histologically proven PCas for initial staging and recurrence detection, (b) having no prior chemotherapy or radiotherapy within 4 weeks before PET imaging. (c) undergoing [^68^Ga]Ga-PSFA-01 and [^68^Ga]Ga-PSMA-11/[^68^Ga]Ga-FAPI-04 scans within 2 weeks. The exclusion criteria were as follows: (a) a time interval between biopsy and PET/CT imaging of less than 2 weeks, (b)had a second primary tumor, (c) patients who were unwilling to provide written informed consent.

### Acquisition of PET/CT images

25, 5, and 3 participants were administered intravenous injections of [^68^Ga]Ga-PSFA-01/[^68^Ga]Ga-PSMA-11, [^68^Ga]Ga-PSFA-01/^68^Ga-FAPI-04, and [^68^Ga]Ga-PSFA-01/[^68^Ga]Ga-PSMA-11/[^68^Ga]Ga-FAPI-04 (2.6-2.8 MBq/kg), respectively. [^68^Ga]Ga-PSFA-01/[^68^Ga]Ga-PSMA-11/[^68^Ga]Ga-FAPI-04 PET/CT were performed using the Philips Gemini TF64 scanner (Philips Medical Systems, USA) 60 min after injection. PET images were acquired in seven bed positions (2 min per bed position). A low-dose CT scan (120 keV, 50 mA) was conducted from the top of the head to the upper thighs. Adverse occurrences and safety information, including blood pressure, heart rate, and temperature, were recorded prior to and 4 hours following the injection of [^68^Ga]Ga-PSFA-01.

### Image interpretation

Visual and semi-quantitative assessments of [^68^Ga]Ga-PSFA-01 PET/CT, [^68^Ga]Ga-PSMA-11 and [^68^Ga]Ga-FAPI-04 PET/CT images were performed independently by two board-certified nuclear medicine physicians (G.L.L. and X.L., with 15 years and 10 years of experience in nuclear medicine, respectively). Each physician conducted an initial independent review of the images. Subsequently, the two physicians met to discuss any discrepancies and reach a consensus on the final interpretation of the images. Reviewers were blinded to the clinical data and other diagnostic information of the patients. In PET/CT assessments, a lesion was considered positive if it demonstrated higher tracer uptake compared to the surrounding background. The results were discussed to reach a consensus in cases of discrepancies. The location and the number of lesions were recorded. Lesions were documented according to their location, including prostate bed, the pelvic and abdominal lymph nodes, bone, lung metastases and metastases in other sites (including cervical and mediastinal lymph nodes, mesorectal metastases). If multiple metastases were identified at a single site, the average standardized uptake value (SUV) was calculated. This was done by averaging the SUVs of all lesions or by calculating the average of the SUVs of the five largest lesions (n > 5). For tumor to background assessment, SUV_max_ values were measured in normal tissues including muscle, kidney, liver, mediastinal blood pool, spleen, pancreas, thyroid, parotid gland, submandibular gland, sublingual gland. The tumor-to-background ratio (TBR) was calculated by dividing the SUV_max_ of the primary tumor by the SUV_mean_ of the mediastinal blood pool. The tumor-to-prostate ratio (TPR) was calculated as the ratio of the primary tumor's SUV_max_ to the SUV_max_ of normal prostate tissue.

A visual scoring system was employed to assess the comparative lesion detection performance of [^68^Ga]Ga-PSFA-01 and [^68^Ga]Ga-PSMA-11 PET. If the area or number of lesions detected by [^68^Ga]Ga -PSFA-01 PET was >1 and <3 times, 3-5 times, or >5 times that detected by [^68^Ga]Ga-PSMA-11 PET, then [^68^Ga]Ga-PSFA-01 PET was scored as 1, 2, and 3, respectively, and vice versa. Notably, when the number or area of lesions detected by the two imaging modalities was identical, the assigned score was 0 24.

### Immunohistochemistry

Pathology sections of PSFA+/PSMA- lesions were collected from one participant's biopsy specimens at the First Affiliated Hospital of Chongqing Medical University. For histopathological analysis, the paraffin-embedded specimens were stained with a fibroblast activation protein alpha Polyclonal antibody (anti-rabbit, diluted to 1:250, Proteintech, BC026250) and a PSMA/GCPII polyclonal antibody (anti-rabbit, diluted to 1:200, Proteintech, BC025672). The sections were dehydrated, sealed, counterstained with hematoxylin, and then examined under a white light microscope. The sections were carefully treated with the DAB chromogenic solution to facilitate visualization; a brown color indicated positive staining.

### Statistical analysis

Statistical analyses were conducted using SPSS software (version 25.0, IBM Inc.). Continuous variables are expressed as mean ± SD. Categorical variables are expressed as numbers or percentages. [^68^Ga]Ga-PSFA-01 and [^68^Ga]Ga-PSMA-11/[^68^Ga]Ga-FAPI-04 uptake and the number of positive lesions were compared using the Wilcoxon signed-rank test. Detection rates were defined as proportions of patients with [^68^Ga]Ga-PSFA-01/[^68^Ga]Ga-PSMA-11/[^68^Ga]Ga-FAPI-04 positive results and were compared by the Mc-Nemar test. A two-tailed P < 0.05 was considered statistically significant.

## Results

### Patient characteristics

**Figure 1:**
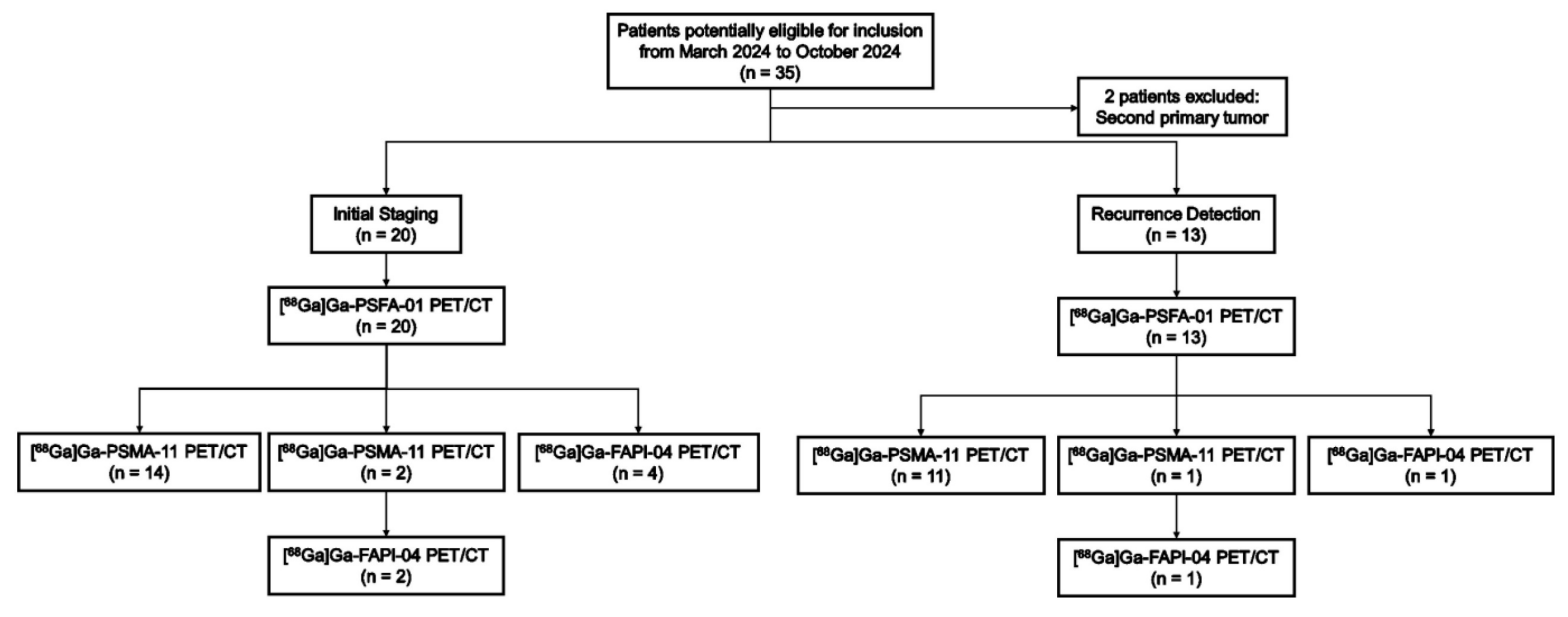
Flow diagram exhibits patient selection details. FAPI: fibroblast activation protein inhibitor. ^68^Ga = gallium 68. PSMA: prostate-specific membrane antigen.

A total of 33 participants (median age, 70 years; range: 52-89 years) were enrolled. Two individuals were not considered eligible for the study because they both had second primary tumors: one suffered from clear cell carcinoma in the right kidney, while the other had a malignant tumor in the ascending colon. Imaging examinations were conducted on 20 patients for initial staging and on 13 patients for recurrence detection. 25 participants underwent both [^68^Ga]Ga-PSFA-01 PET/CT and [^68^Ga]Ga-PSMA-11 PET/CT scans, 5 participants underwent both [^68^Ga] Ga-PSFA PET/CT and [^68^Ga]Ga-FAPI-04 PET/CT scans and 3 participants underwent [^68^Ga]Ga-PSFA-01 PET/CT, [^68^Ga]Ga-PSMA-11 PET/CT and [^68^Ga]Ga-FAPI-04 PET/CT scans within 2 weeks **(Figure 1)**. Among the 33 patients, 30 patients demonstrated positive lesions while the other 3 patients demonstrated negative findings in both [^68^Ga]Ga-PSFA-01 and [^68^Ga]Ga-PSMA-11 PET/CT. No adverse events were observed or reported in any participant during the radiopharmaceutical administration of [^68^Ga]Ga-PSFA-01. Patient characteristics are listed in **Table 1**.

### Comparison of [^68^Ga]Ga-PSFA-01 and [^68^Ga]Ga-PSMA-11

#### Comparison of SUV_max_

Compared to [^68^Ga]Ga-PSMA-11, [^68^Ga]Ga-PSFA-01 demonstrated significantly lower uptake in primary tumors (11.13 ± 7.04 vs. 15.44 ± 9.25, p = 0.009), bone metastases (8.50 ± 5.0 vs. 12.43 ± 9.55, p < 0.001) and metastases in other sites (6.05 ± 3.29 vs. 10.73 ± 8.74, p = 0.028). Meanwhile, SUV_max-PSFA_ was lower than SUV_max-PSMA_ in pelvic lymph nodes (9.17 ± 4.89 vs. 12.96 ± 6.38, p = 0.077), abdominal lymph nodes (7.68 ± 4.29 vs. 14.71 ± 10.55, p = 0.056) and pulmonary metastases (3.34 ± 0.41 vs. 3.86 ± 1.34, p = 0.18). Additionally, [^68^Ga]Ga-PSFA-01 PET/CT exhibited lower TBR (2.86 ± 1.50 vs. 9.50 ± 5.62, p < 0.001).Tumor-to-prostate (TPR) was also compared, but no statistically significant difference was observed (6.92 ± 4.90 vs. 7.95 ± 5.62, p = 0.624). The comparative uptake of [^68^Ga]Ga-PSFA-01 and [^68^Ga]Ga-PSMA-11 in PCa is delineated in **Table 2**.

In normal tissues, [^68^Ga]Ga-PSFA-01 demonstrated significantly higher background activities in the thyroid (SUV_max_, 11.39 ± 2.76 vs. 1.49 ± 0.49) and pancreas (SUV_max_, 9.90 ± 2.53 vs. 2.15 ± 0.72) (**Table S1**).

#### Comparison of visual assessment

**Figure 2:**
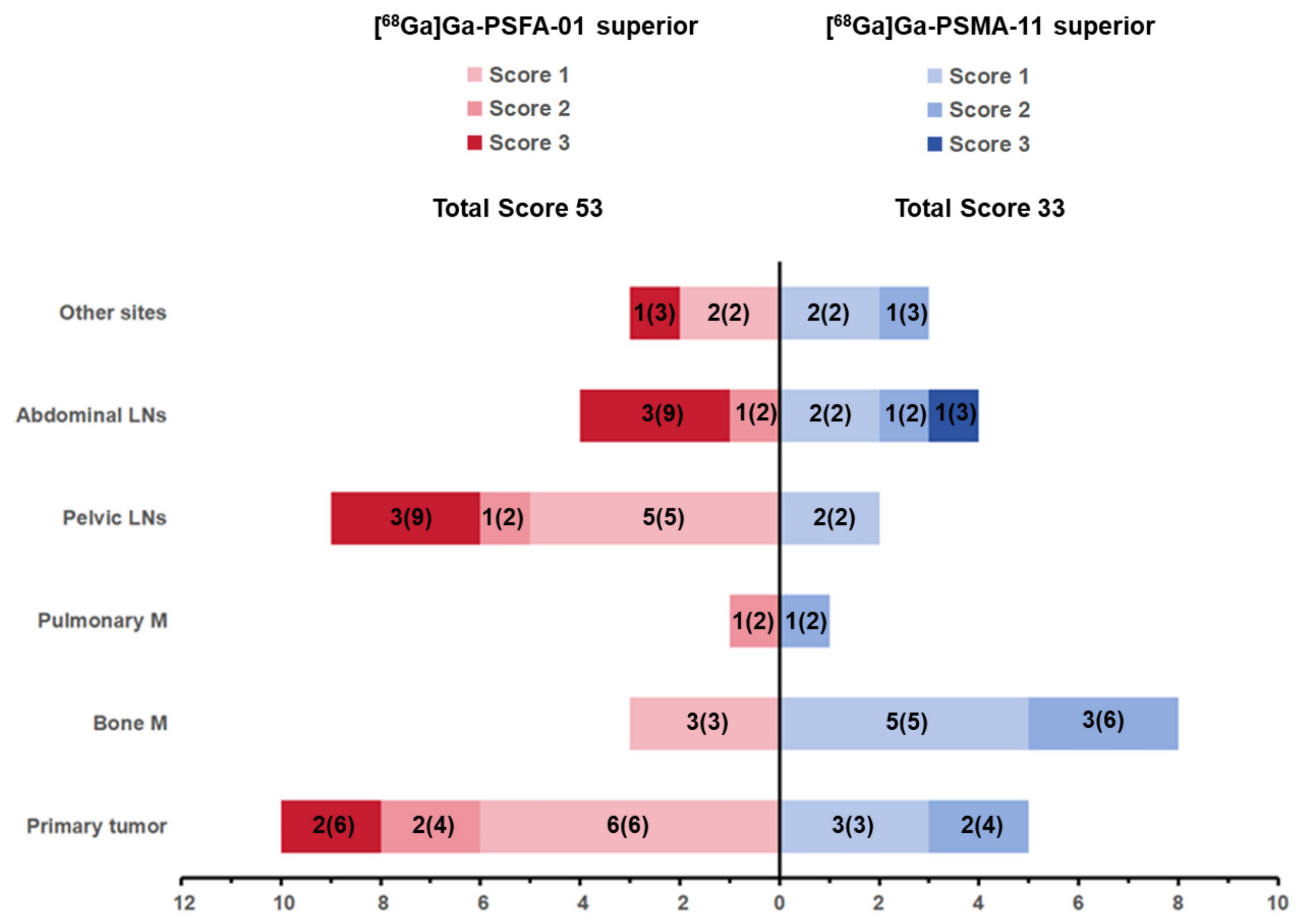
Comparison of visual assessment between [^68^Ga]Ga-PSFA-01 PET and [^68^Ga]Ga-PSMA-11 PET, n (n) within each bar denotes the patient number (scores).

The detectability of [^68^Ga]Ga-PSFA-01 was markedly superior to that of [^68^Ga]Ga-PSMA-11 PET, with the former being awarded a considerably higher overall score (53 vs. 33). Specifically, [^68^Ga]Ga-PSFA-01 PET demonstrated a significant advantage compared to [^68^Ga]Ga-PSMA-11 PET, identifying a substantially greater number or larger lesions in various sites, including primary tumor (Scores 16 vs. 7, Equal: 7), pelvic lymph nodes (Scores 16 vs. 2, Equal: 4), abdominal lymph nodes (Scores 11 vs. 7, Equal: 2) and metastases in other sites (Scores 11 vs. 7, Equal: 2). The visual assessment is shown in** Figure 2**.

### Patient-based analysis of [^68^Ga]Ga-PSFA-01 and [^68^Ga]Ga-PSMA-11 PET/CT

**Figure 3:**
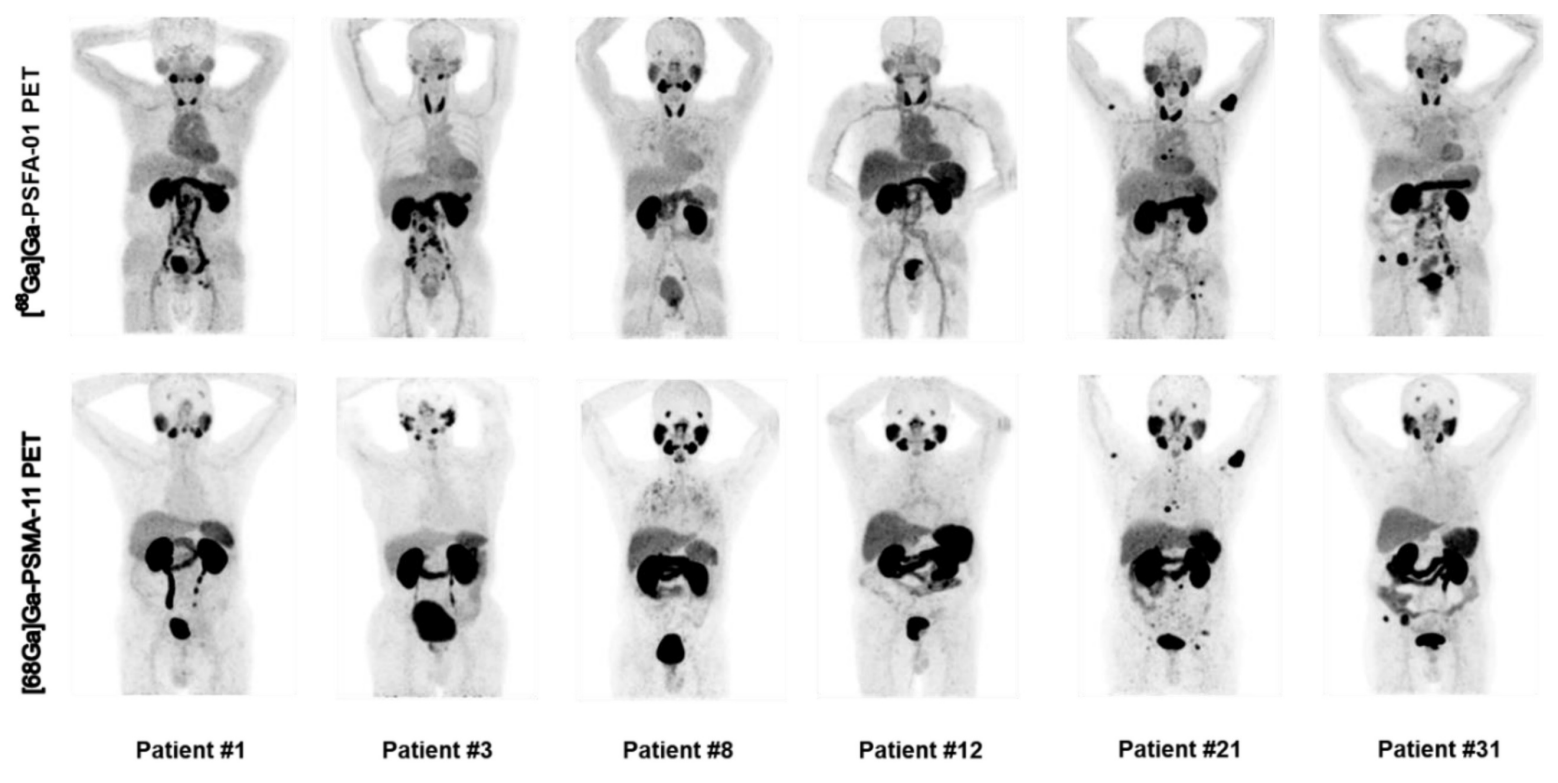
Representative images of [^68^Ga]Ga-PSFA-01 PET and [^68^Ga]Ga-PSMA-11 PET in patients with PCa for initial staging (patients #1, #3, #8, and #12) and recurrence detection (patients #21 and #31).

For patient-based analysis of detection rates, [^68^Ga]Ga-PSFA-01 was superior to [^68^Ga]Ga-PSMA-11 in depicting pelvic lymph nodes (50.0% [14/28] vs. 46.4% [13/28], p > 0.5), abdominal lymph nodes (67.9% [19/28] vs. 57.1% [16/28], p = 0.25) and metastases in other sites (35.7% [10/28] vs. 25.0% [7/28], p = 0.25). Both imaging modalities exhibited identical detection rates for primary prostate lesions (82.1% [23/28] vs. 82.1% [23/28]), bone metastases (57.1% [16/28] vs. 57.1% [16/28]), and pulmonary metastases (7.1% [2/28] vs. 7.1% [2/28]). **Figure 3** illustrates the maximum intensity projection images obtained from two distinct imaging studies for six patients.

### Lesion-based analysis of [^68^Ga]Ga-PSFA-01 and [^68^Ga]Ga-PSMA-11 PET/CT

**Figure 4:**
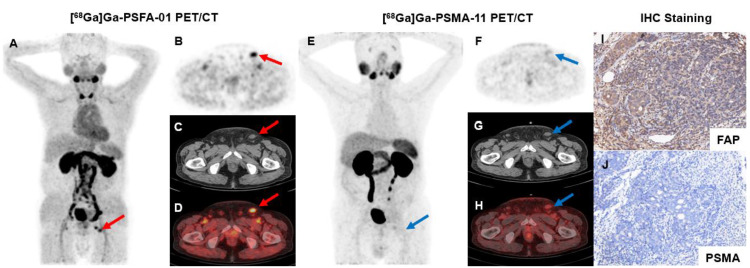
A 71-year-old man (Patient #1) was diagnosed with metastatic PCa through aspiration of the left inguinal mass. The patient's current serum tPSA level was 86.08 ng/mL. The SUVmax of dual-target tracer [^68^Ga]Ga-PSFA-01 (A-D, red arrows) was visually higher than that of the single-target tracer [^68^Ga]Ga-PSMA-11 (E-H, blue arrows) in the left inguinal lymph nodes (SUVmax, 7.77 vs. 2.69). Immunohistochemical staining showed positively high expression of FAP and negative expression of PSMA (I, J).

**Figure 5:**
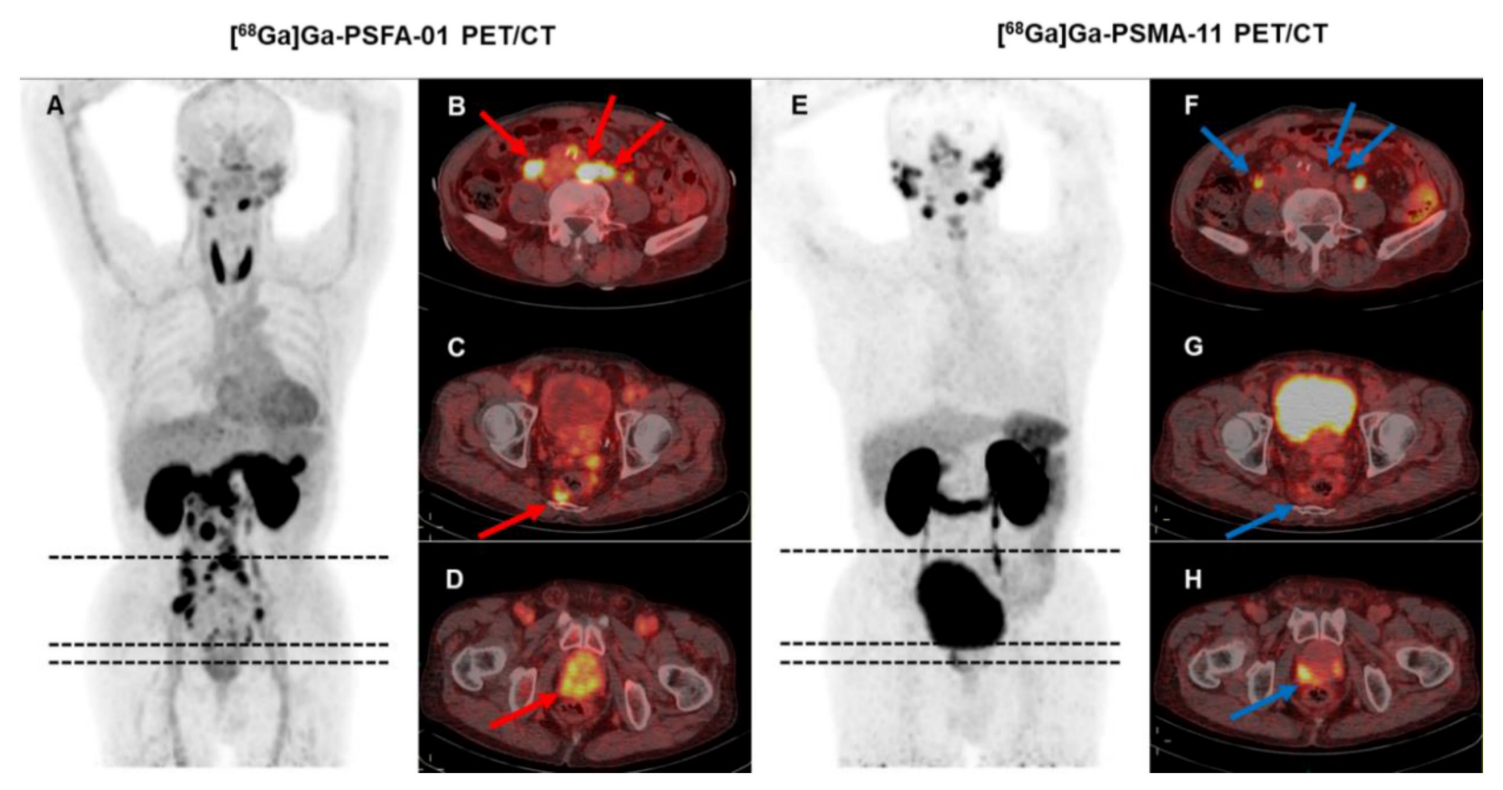
A 74-year-old man (Patient #3) with a Gleason score of 4+4 and a current serum tPSA level of 86.00 ng/mL. [^68^Ga]Ga-PSFA-01 PET/CT showed a greater number of lesions (A-D, red arrows) and more intense tracer uptake than [^68^Ga]Ga-PSMA-11 PET/CT (E-H, blue arrows). In [^68^Ga]Ga-PSFA-01 PET/CT scans, para-abdominal lymph nodes and mesenteric metastases exhibited positive results (B, C), whereas in [^68^Ga]Ga-PSMA-11 PET/CT scans, these findings were absent (F, G).

[^68^Ga]Ga-PSFA-01 PET/CT detected a significantly higher number of prostate lesions compared to [^68^Ga]Ga-PSMA-11 PET/CT (323 vs. 265, p < 0.001). For lesion-based analysis, [^68^Ga]Ga-PSFA-01 imaging identified a significantly higher number of pelvic lymph nodes (55 vs. 29, p < 0.001), abdominal lymph nodes (36 vs. 19, p < 0.001), and bone metastases (162 vs. 156, p = 0.195) compared to [^68^Ga]Ga-PSMA-11 PET. Additionally, it detected a non-significantly higher number of primary lesions (49 vs. 42, p = 0.18), lung metastases (3 vs. 2, p = 0.716) and a similar number of metastases at other sites (18 vs. 17, p = 0.716). **Figures 4-5** display the representative image.

### Subgroup analysis of [^68^Ga]Ga-PSFA-01 with [^68^Ga]Ga-PSMA-11 PET/CT

We divided the patients into two subgroups for analysis: initial staging (n = 20) and recurrence detection (n = 13). In each subgroup, we assessed and compared the uptake of [^68^Ga]Ga-PSFA-01 and [^68^Ga]Ga-PSMA-11 PET/CT in lesions from different locations. In patients undergoing initial staging, [^68^Ga]Ga-PSFA-01 exhibited lower uptake compared to [^68^Ga]Ga-PSMA-11 in primary tumors (12.49 ± 7.70 vs. 15.95 ± 9.91, p = 0.079), bone metastases (12.28 ± 7.64 vs. 25.29 ± 15.35, p = 0.002), pelvic lymph nodes (10.99 ± 5.72 vs. 13.97 ± 5.78, p = 0.182), abdominal lymph nodes (8.48 ± 5.44 vs. 15.80 ± 11.8, p = 0.158) and metastases in other sites (4.23 ± 1.46 vs. 7.37 ± 4.43, p = 0.05). In patients with recurrence detection, [^68^Ga]Ga-PSFA-01 demonstrated lower uptake in primary tumors (8.44 ± 4.76 vs. 14.1 ± 7.68, p = 0.013) , bone metastases (8.54 ± 5.02 vs. 12.07 ± 9.04, p < 0.001), pelvic lymph nodes (6.86 ± 3.40 vs. 11.57 ± 7.29, p = 0.327), abdominal lymph nodes (6.48 ± 1.51 vs. 11.44 ± 4.91, p = 0.068) and pulmonary metastases (3.11 ± 0.49 vs. 3.61 ± 1.05, p = 0.109),and metastases in other sites (9.06 ± 3.70 vs. 16.89 ± 11.63, p = 0.05) compared to [^68^Ga]Ga-PSMA-11 (Tables 3-4).

### Comparison of [^68^Ga]Ga-PSFA-01 with [^68^Ga]Ga-FAPI-04 PET/CT

**Figure 6:**
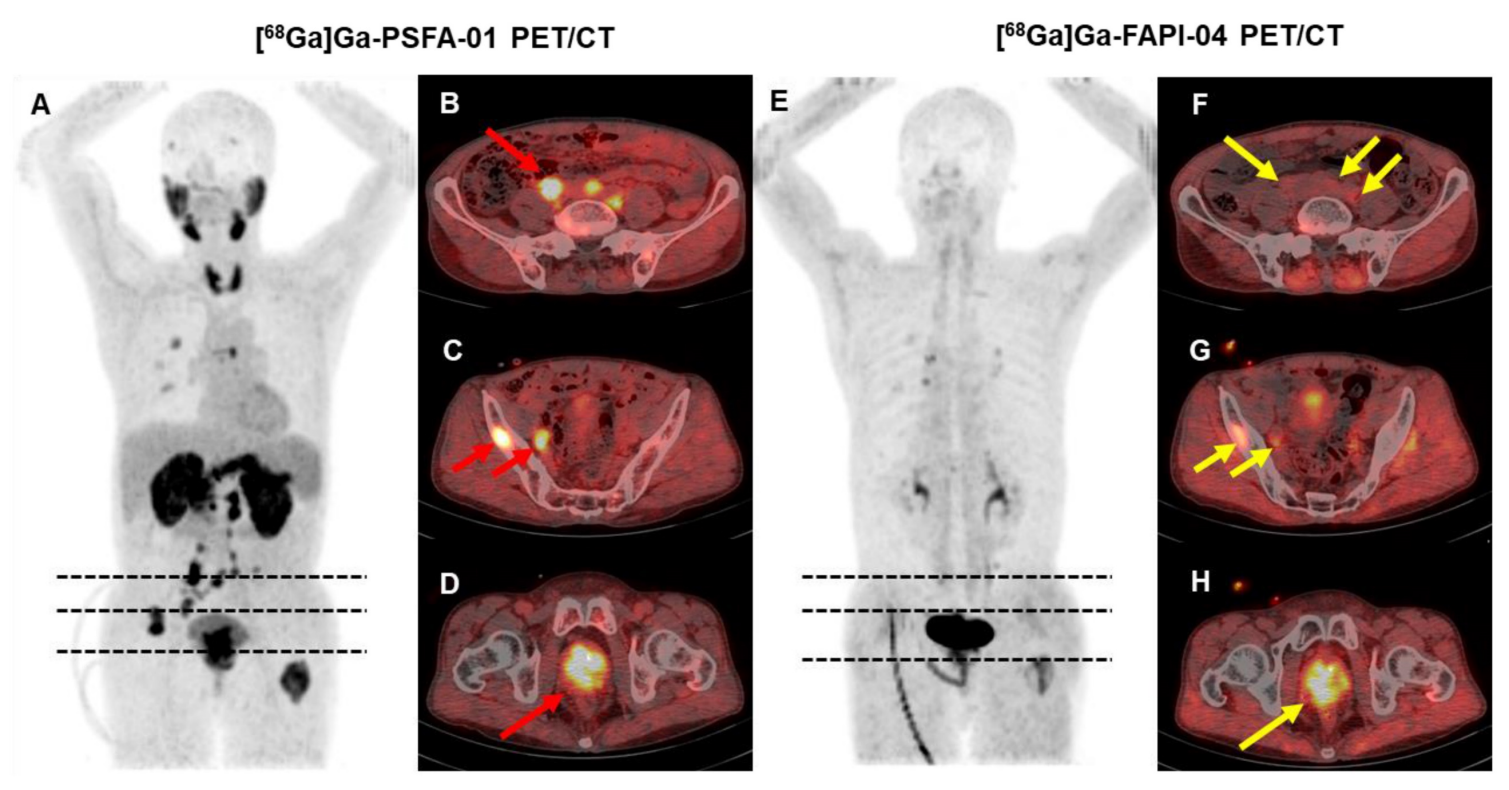
A 66-year-old man (Patient #18) with a Gleason score of 4+3 and a current serum tPSA level of 263.0 ng/mL. [^68^Ga]Ga-PSFA-01 PET/CT imaging revealed the presence of positive lesions in bilateral para-iliac artery and right obturator region (A-D, red arrows), which were not as distinctly evident on [^68^Ga]Ga-FAPI-04 PET/CT (E-H, yellow arrows).

We conducted both [^68^Ga]Ga-PSFA-01 and [^68^Ga]Ga-FAPI-04 on 5 participants. [^68^Ga]Ga-PSFA-01 PET/CT detected more lesions than [^68^Ga]Ga-FAPI (24 vs. 13, p = 0.18). Higher SUVmax were noted for [^68^Ga]Ga-PSFA-01 PET/CT scan compared with [^68^Ga]Ga-FAPI-04 PET/CT (primary tumors, 10.27 ± 2.42 vs. 7.32 ± 0.17, p = 0.109; bone metastases, 8.14 ± 5.98 vs. 4.52 ± 1.22, p = 0.128; pelvic lymph nodes, 5.4 ± 2.83 vs. 4.19 ± 1.39, p = 0.655), although the differences were not statistically significant. **Figure 6** shows a typical image.

### Comparison of [^68^Ga]Ga-PSFA-01 with [^68^Ga]Ga-PSMA-11 and [^68^Ga]Ga-FAPI-04 PET/CT

For comparative analysis with [^68^Ga]Ga-PSFA-01 PET/CT, we enrolled 3 participants (#11, #12, #31) who underwent PET/CT scans with both dual-target and two different single-target tracers. Uptake of [^68^Ga]Ga-PSFA-01 was lower than that of [^68^Ga]Ga-PSMA-11, while [^68^Ga]Ga-FAPI-04 exhibited the lowest uptake (primary tumor, SUV_max-PSFA_ vs. SUV_max-PSMA_ vs. SUV_max-FAPI_, 16.27 ± 4.34 vs. 24.07 ± 10.55 vs. 13.03 ± 5.03, bone metastases, 8.60 ± 5.61 vs. 9.72 ± 5.84 vs. 8.2 ± 5.23). In detecting metastatic lesions, [^68^Ga]Ga-PSFA-01 demonstrates superior performance compared to [^68^Ga]Ga-PSMA-11 and [^68^Ga]Ga-FAPI-04 (34 vs. 25 vs. 13). In #31 patient, [^68^Ga]Ga-PSFA-01 and [^68^Ga]Ga-FAPI-04 identified metastatic para-abdominal aortic lymph nodes, whereas [^68^Ga]Ga-PSMA-11 failed to detect them. However, in #11 patient, both [^68^Ga]Ga-PSFA-01 and [^68^Ga]Ga-PSMA-11 detected metastatic pelvic lymph nodes, which were negative in [^68^Ga]Ga-FAPI-04. 1 of the 3 patients have been previously reported 23 (**Figure S1**).

### Correlation between [^68^Ga]Ga-PSFA-01 PET/CT and patients' characteristics

The SUV_max-PSFA_ of prostate lesions exhibits a significant positive correlation with tPSA levels (r = 0.468, p = 0.016) and fPSA levels (r = 0.518, p = 0.04), a significant negative correlation with FPSAR (r = -0.608, p = 0.012), and a positive correlation with Gleason score (r = 0.086, p = 0.705) and WHO/ISUP grade group (r = 0.055, p = 0.808).

## Discussion

PSMA PET imaging has been widely utilized in the management of PCa, highly regarded for its high sensitivity and specificity. It has higher diagnostic performance than traditional imaging, especially in the initial staging and localization of high-risk PCa 25,26. However, the application of PSMA PET also has limitations. In cases where individuals with PCa undergo disease progression during therapy, PSMA PET/CT imaging may demonstrate unexpected outcomes. This is often attributed to the fluctuations in PSA levels, which can impact the accuracy and interpretation of the imaging results 27. In addition, not all PCa cells overexpress PSMA. In PSMA positive tumors, only 20-80% of PCa cells are immunohistochemically positive. The heterogeneity of tumors may lead to false-negative results in PSMA PET 28. To overcome these limitations, several alternative tracers are therefore being investigated. For example, choline tracers such as [^18^F]Fluoromethylcholine are utilized to assess phospholipid metabolism, whereas DOTA-peptide PET agents exhibit heightened sensitivity to tumor neuroendocrine differentiation. Both have shown potential in patients with tumor dedifferentiation or neuroendocrine changes after multiple treatments 29-31. In addition, prior research indicates that dual-targeting PET imaging tracers offer benefits such as enhanced tumor uptake, improved diagnostic precision, reduced effects of tumor heterogeneity, increased receptor-ligand affinity, and better tumor specificity, while also exhibiting good pharmacokinetic properties 32-34.

In this prospective clinical study, we initially conducted a comparative analysis between the [^68^Ga]Ga-PSFA-01, [^68^Ga]Ga-PSMA-11, and [^68^Ga]Ga-FAPI-04 PET scans for the detection of primary and metastatic lesions in PCa. Our results indicate that [^68^Ga]Ga-PSFA-01 PET possesses significant diagnostic utility in the identification of PCa.

In patient-based analyses between [^68^Ga]Ga-PSFA-01 and [^68^Ga]Ga-PSMA-11 PET/CT, [^68^Ga]Ga-PSFA-01 demonstrated superior performance in visualizing pelvic and abdominal lymph nodes, as well as metastases at other sites, including cervical and mediastinal lymph nodes and mesorectal metastases. However, for the detection of primary lesions and bone metastases, both tracers exhibited comparable efficacy. Furthermore, in lesion-based analysis, [^68^Ga]Ga-PSFA-01 imaging identified a significantly higher number of primary and metastatic lesions in PCa compared to other modalities. The findings highlight the additional diagnostic value of dual-target imaging agents in the detection of PCa lesions. The dual-target nature of [^68^Ga]Ga-PSFA-01, which targets both PSMA and FAP, is a key feature that enhances its detection rates. PSMA is highly expressed in PCa cells, while FAP is overexpressed in CAFs within the tumor microenvironment. By targeting FAP, [^68^Ga]Ga-PSFA-01 can potentially reveal additional information about the tumor microenvironment that may not be evident with PSMA targeting alone. This dual-targeting approach allows for more comprehensive imaging of both the primary tumor and the surrounding stromal cells, which play a significant role in tumor progression.

It was observed that lesions exhibited a higher uptake with [^68^Ga]Ga-PSMA-11 than with [^68^Ga]Ga-PSFA-01 PET/CT, and this finding was also confirmed in the subgroup analysis. Verena A *et al.* previously synthesized three imaging agents capable of targeting both PSMA and FAPI, [^68^Ga]Ga-AV01017, [^68^Ga]Ga-AV01030 and [^68^Ga]Ga-AV01038, which exhibited low uptake in PCa lesions 35. With the aim of enhancing tumor uptake and the quality of imaging, the team has reformulated two PSMA/FAP-targeted tracers, [^68^Ga]Ga-AV01084 and [^68^Ga]Ga-AV01088. Despite modest enhancements in tumor uptake, dual-targeting tracers still exhibit lower uptakes compared to single-targeting tracers 36. Accordingly, while dual-target tracers have successfully enhanced lesion detection rates, further optimization of radiopharmaceutical structures is essential to achieve increased tumor lesion uptake. It is noteworthy that [^68^Ga]Ga-PSFA-01 outperforms other tracers in terms of lesion detection and visual analysis. This highlights the importance of assessing the efficacy of a PET imaging agent by taking into account a range of factors, including diagnostic accuracy, lesion detection capabilities, specificity and sensitivity, radiation dosage and safety, image quality, stability, biological distribution, and clinical application potential.

The overexpression of FAP in CAFs has been shown to drive an immunosuppressive and growth-promoting microenvironment in various cancers, including PCa. A study evaluated FAP expression across different clinical stages of PCa and explored the potential application of [^68^Ga]Ga-FAPI-04 PET/CT imaging in CRPC. The study found that the average H-Index values for benign prostate tissue, primary PC, neoadjuvant androgen deprivation therapy before radical prostatectomy, CRPC, and neuroendocrine prostate cancer (NEPC) were 0.018, 0.031, 0.042, 0.076, and 0.051, respectively. These results indicate a significant increase in FAP expression with disease progression. Additionally, in three patients who underwent [^68^Ga]Ga-FAPI-04 PET/CT, highly positive PET signals were observed, with multiple metastatic lesions detected. This study provides a robust foundation for further research into the application of FAPI in PCa 37. Meanwhile, the expression of FAP receptors in PCa, as demonstrated in previous studies, was also observed in our study 17,20. In patient #1, [^68^Ga]Ga-PSFA-01 PET/CT identified a positive lesion in the left inguinal region, contrasting with the negative result observed in [^68^Ga]Ga-PSMA-11 PET. Subsequent immunohistochemical analysis confirmed FAP positivity and PSMA negativity in the inguinal mass, underscoring the significance of developing dual-target imaging agents for diagnostic purposes.

In recent years, the integration of therapeutic isotopes labeled with PSMA receptors has emerged as a prominent research area for the treatment of mCRPC 38,39. Notably, [^177^Lu]Lu-PSMA-617, when used alone or in conjunction with enzalutamide, docetaxel, and other agents, has demonstrated high response rates, low toxicity, and effective pain relief in mCRPC patients 40. With the increased detection efficiency of dual-target agents on tumor lesions, there is a growing anticipation for the future development of dual-target probes that could significantly impact the clinical diagnosis and treatment of mCRPC patients.

There are several limitations as well. First, the diagnostic significance of the PET tracer may not have been adequately assessed due to the small number of participants. Second, the diverse manifestations of PCa at various stages on [^68^Ga]Ga-PSFA-01 PET/CT and other PET tracers. Consequently, there is a clear need for an increased patient population to enable thorough subgroup analysis and comparative studies. Third, a lack of tissue samples resulted in incomplete immunohistochemical validation, potentially affecting the study's outcomes. Therefore, to verify the accuracy of the results, future studies must include more pathological samples. Lastly, due to incomplete clinical data on treatment details, we could not further assess the correlation between [^68^Ga]Ga-PSFA-01 imaging with hormone therapy status. This limitation highlights the need for more complete datasets in future studies to better understand these relationships. Furthermore, subsequent studies may prioritize the investigation of radiolabeled dual-target radiopharmaceuticals in the context of therapeutic applications, which could potentially lead to more effective treatment strategies. Furthermore, it is crucial to investigate the potential applications of [^68^Ga]Ga-PSFA-01 PET/CT in a wider range of tumor types to confirm its effectiveness and therapeutic potential.

## Conclusion

Our investigation utilized PET/CT scans with the dual-target tracer [^68^Ga]Ga-PSFA-01 to identify PCa lesions and conducted an initial comparative study with [^68^Ga]Ga-PSMA-11 PET/CT and [^68^Ga]Ga-FAPI-04 PET/CT. [^68^Ga]Ga-PSFA-01 PET/CT exhibited better lesion detection efficiency for primary and metastatic PCa lesions, surpassing the performance of the other two imaging agents. Further pathological investigations are needed to substantiate the findings of this study.

## Supplementary Material

Supplementary figure and table.

## Figures and Tables

**Table 1:** Patient characteristics

No.	Age(y)	tPSA (ng/mL)	fPSA (ng/mL)	FPSAR	WHO/ISUP grade group	Gleason score	Role of PET	Therapy	[^68^Ga]Ga-PSFA-01	[^68^Ga]Ga-PSMA-11	[^68^Ga]Ga-FAPI-04
1	71	86.08	10.16	0.12		*	IS		P	P	
2	72	55.53	2.03	0.04		*	IS		P	P	
3	74	86.00			4	4+4=8	IS		P	P	
4	52	36.04			5	4+5=9	IS		P	P	
5	62	0.09	0.02	0.22	5	4+5=9	IS		P	P	
6	72	2837.00	101.6	0.04	5	4+5=9	IS		P	P	
7	71	175.30			5	5+4=9	IS		P	P	
8	54	100.00			4	4+4=8	IS		P	P	
9	69	153.10	10.00	0.07	5	4+5=9	IS		P	P	
10	76	0.01	<0.01		3	4+3=7	IS		P	P	
11	68	132.80			4	5+3=8	IS		P	P	P
12	76	170.70	17.06	0.10	2	3+4=7	IS		P	P	P
13	65	6.76	1.08	0.16	5	5+4=9	IS		P	P	
14	62	261.10	30.09	0.12	5	5+4=9	IS		P	P	
15	70	785.40	53.20	0.07	4	4+4=8	IS		P	P	
16	59	78.14			3	4+3=7	IS		P	P	
17	73	11.90			2	3+4=7	IS		P		P
18	66	263.00	25.70	0.10	3	4+3=7	IS		P		P
19	69	46.70	7.45	0.16	4	4+4=8	IS		P		P
20	81	29.04	3.52	0.12	5	4+5=9	IS		P		P
21	67	4.35	0.96	0.22		*	RD	Radiation therapy+ ADT	P	P	
22	55	0.01	0.01	1.00	4	3+5=8	RD	Prostatectomy+ ADT	N	N	
23	70	16.38	5.90	0.36	4	4+4=8	RD	ADT	P	P	
24	69	1.49	0.12	0.08	2	3+4=7	RD	Prostatectomy+ Salvage radiation therapy+ ADT	N	N	
25	71	1.70			1	2+3=5	RD	Prostatectomy+ Radiation therapy +ADT	P	P	
26	84	0.01	0.01	1.00	2	3+4=7	RD	Prostatectomy +ADT	P	P	
27	84	1.91	0.30	0.16	4	5+3=8	RD	ADT	P	P	
28	89	698.80	47.19	0.07	5	5+4=9	RD	ADT	P	P	
29	76	7.91	0.97	0.12	1	2+3=5	RD	Prostatectomy+ ADT	P	P	
30	58	1.83	0.20	0.11	4	3+5=8	RD	Prostatectomy+ Radiation therapy +ADT	N	N	
31	73	52.99	2.43	0.05	5	4+5=9	RD	Chemotherapy +ADT	P	P	P
32	58	17.03	2.38	0.14	2	3+4=7	RD	ADT	P		P
33	78	7.23				*	RD	Prostatectomy +ADT	P	P	

ADT: Androgen deprivation therapy. fPSA: Free Prostate-Specific Antigen, FPSAR: Free-to-Total Prostate-Specific Antigen Ratio. IS: Initial staging. N: Negative. P: Positive. RD: Recurrence detection. tPSA: Total Prostate-Specific Antigen, WHO/ISUP: World Health Organization/International Society of Urological Pathology. * #1 patient's pathological biopsy diagnosed enlarged lymph nodes in the right inguinal region as metastatic PCa. #2, #33 patients were diagnosed with metastatic PCa through left pelvic wall lymph node biopsy. #21 patient was diagnosed with metastatic PCa through left cervical lymph node biopsy. No treatment measures were implemented by the patient for initial staging.

**Table 2:** Comparison between [^68^Ga]Ga-PSFA-01 and [^68^Ga]Ga-PSMA-11 Uptake in PCa

	[^68^Ga]Ga-PSFA-01 PET/CT		[^68^Ga]Ga-PSMA-11 PET/CT		
Lesion location	No. of lesions	SUV_max_	No. of lesions	SUV_max_	P value
Primary tumor	49	11.13 ± 7.04	42	15.44 ± 9.25	0.009
Bone M	162	8.50 ± 5.0	156	12.43 ± 9.55	< 0.001
Pelvic LNs	55	9.17 ± 4.89	29	12.96 ± 6.38	0.077
Abdominal LNs	36	7.68 ± 4.29	19	14.71 ± 10.55	0.056
Pulmonary M	3	3.34 ± 0.41	2	3.86 ± 1.34	0.18
Other sites	18	6.05 ± 3.29	17	10.73 ± 8.74	0.028

LNs: lymph nodes, M: metastases, SUVmax: maximum standardized uptake value.

**Table 3:** Comparison between [^68^Ga]Ga-PSFA-01 and [^68^Ga]Ga-PSMA-11 Uptake in initial staging detection

Lesion location	SUV_max-PSFA_	SUV_max-PSMA_	P value
Primary tumor	12.49 ± 7.70	15.95 ± 9.91	0.079
Bone M	12.28 ± 7.64	25.29 ± 15.35	0.002
Pelvic LNs	10.99 ± 5.72	13.97 ± 5.78	0.182
Abdominal LNs	8.48 ± 5.44	15.80 ± 11.8	0.158
Other sites	4.23 ± 1.46	7.37 ± 4.43	0.05

**Table 4:** Comparison between [^68^Ga]Ga-PSFA-01 and [^68^Ga]Ga-PSMA-11 Uptake in detecting recurrence

Lesion location	SUV_max-PSFA_	SUV_max-PSMA_	P value
Primary tumor	8.44 ± 4.76	14.1 ± 7.68	0.013
Bone M	8.54 ± 5.02	12.07 ± 9.04	< 0.001
Pelvic LNs	6.86 ± 3.40	11.57 ± 7.29	0.327
Abdominal LNs	6.48 ± 1.51	11.44 ± 4.91	0.068
Pulmonary M	3.11 ± 0.49	3.61 ± 1.05	0.109
Other sites	9.06 ± 3.70	16.89 ± 11.63	0.05

## Abbreviations

CAFs: cancer-associated fibroblasts
CRPC: castration-resistant prostate cancer
FAP: fibroblast activation protein
FAPI: FAP inhibitor
mCRPC: metastatic castration-resistant prostate cancer
PCa: prostate cancer
PET: positron emission tomography
PSMA: prostate-specific membrane antigen
SUV_max_: maximum standardized uptake
TBR: tumor to background ratio
TPR: tumor to prostate ratio
