# Supplementary material for: Dual targeting PET tracer [68Ga]Ga-PSFA-01 in patients with prostate cancers: A pilot exploratory study
Source: Theranostics. 2025 Mar 10;15(9):4124–34. doi: 10.7150/thno.108676 (PMC11980653; doi:10.7150/thno.108676)
Supplement: Supplementary file 1 — Supplementary figure and table. [file thnov15p4124s1.pdf]

Supplementary table 1. SUVmax of [^{68}Ga]Ga-PSFA-01, [^{68}Ga]Ga-PSMA-11, and [^{68}Ga]Ga-FAPI-04 in Normal Tissue

	[^{68}Ga]Ga-PSFA-01	[^{68}Ga]Ga-PSMA-11	[^{68}Ga]Ga-FAPI-04
Muscle	1.43 ± 0.29	0.63 ± 0.18	1.63 ± 0.36
Kidney	12.22 ± 3.38	39.12 ± 12.99	2.08 ± 0.35
Liver	3.83 ± 0.83	4.79 ± 1.31	1.03 ± 0.25
Mediastinal blood pool	3.78 ± 0.72	1.62 ± 0.36	1.58 ± 0.30
Spleen	4.83 ± 0.96	7.15 ± 1.95	1.05 ± 0.24
Pancreas	9.90 ± 2.53	2.15 ± 0.72	1.55 ± 0.35
Thyroid	11.39 ± 2.76	1.49 ± 0.49	1.49 ± 0.52
Parotid gland	5.60 ± 1.53	11.36 ± 4.01	1.26 ± 0.39
Submandibular gland	9.19 ± 2.38	13.86 ± 4.37	2.69 ± 1.09
Sublingual gland	4.85 ± 1.66	5.52 ± 3.06	2.11 ± 0.74

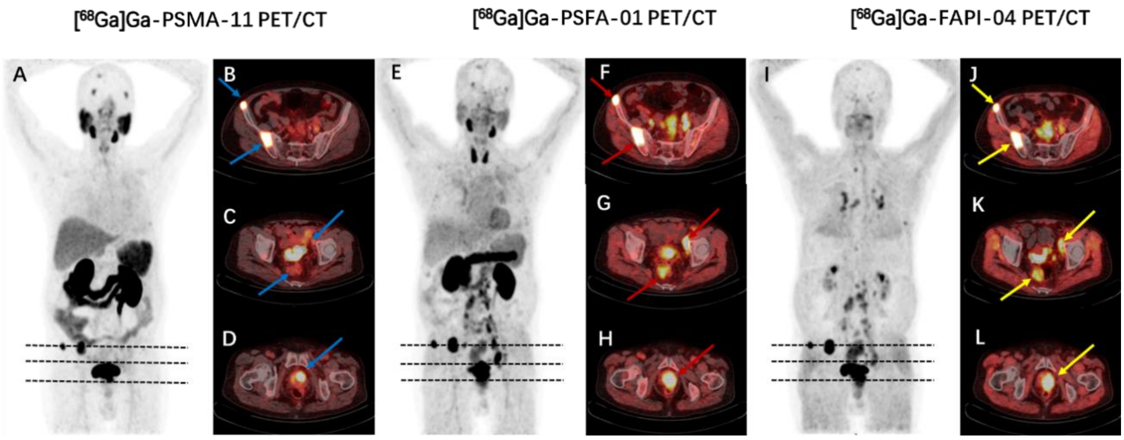

Supplementary figure 1. A 73-year-old man with a Gleason score of 4+5 and current serum tPSA level of 52.99 ng/ml underwent both dual-targeting and single-targeting imaging. (**A, B, C**) The three imaging techniques identified metastatic lesions in the right iliac bone and primary prostate cancer. Nevertheless, only [^{68}Ga]Ga-PSFA-01 and [^{68}Ga]Ga-FAPI-04 PET/CT revealed positive lymph nodes in the left obturator area and mesorectal metastases.
